# Cross-Protection Induced by a A/MAY/97 Emergency Vaccine Against Intra-Serotype Heterologous Challenge with a Foot-and-Mouth Disease Virus from the A/ASIA/G-VII Lineage

**DOI:** 10.3390/vaccines8010024

**Published:** 2020-01-14

**Authors:** Aldo Dekker, Beatriz Sanz-Bernardo, Nagendrakumar Balasubramanian Singanallur, Anna B. Ludi, Jacquelyn Horsington, Phaedra L. Eblé, Donald P. King, Wilna Vosloo

**Affiliations:** 1 Wageningen Bioveterinary Research, P.O. Box 65, 8200 AB Lelystad, The Netherlands; Phaedra.Eble@wur.nl; 2 The Pirbright Institute, Ash Road, Pirbright Surrey GU24 0NF, UK; Beatriz.Sanz-Bernardo@pirbright.ac.uk (B.S.-B.); Anna.Ludi@pirbright.ac.uk (A.B.L.); Donald.King@pirbright.ac.uk (D.P.K.); 3 CSIRO-Australian Animal Health Laboratory, Private bag 24, Geelong 3220, Australia; Nagendra.Singanallur@csiro.au (N.B.S.); Jacquelyn.Horsington@merck.com (J.H.); Wilna.Vosloo@csiro.au (W.V.); 4 Intervet International BV, Wim de Körverstraat 35, P.O. Box 31, 5830 AA Boxmeer, The Netherlands

**Keywords:** FMD, foot-and-mouth disease, vaccine, cross-protection, heterologous protection, potency test

## Abstract

Since 2015, outbreaks of foot-and-mouth disease (FMD) in the Middle East have been caused by a new emerging viral lineage, A/ASIA/G-VII. *Invitro* vaccine matching data indicated that this virus poorly matched (low r_1_-value) with vaccines that were being used in the region as well as most other commercially available vaccines. The aim of this study was to assess the performance of two candidate vaccines against challenge with a representative field virus from the A/ASIA/G-VII lineage. The results from an initial full dose protection study provided encouraging data for the A/MAY/97 vaccine, while the A_22_/IRQ/64 vaccine only protected 2/7 vaccinated animals. In view of these promising results, this vaccine was tested in a potency test (PD_50_) experiment in which 5 cattle were vaccinated with a full dose, 5 cattle with a 1/3 dose and 5 cattle with a 1/9 dose of vaccine. At 21 days post vaccination these vaccinated cattle and 3 control cattle were challenged intradermolingually with a field isolate from the A/ASIA/G-VII lineage. The intra-serotype heterologous potency test resulted in an intra-serotype heterologous potency of 6.5 PD_50_/dose. These data support previous studies showing that a high potency emergency vaccine can protect against clinical disease when challenged with a heterologous strain of the same serotype, indicating that not only the r_1_-value of the vaccine, but also the homologous potency of a vaccine should be taken into account when advising vaccines to control an outbreak.

## 1. Introduction

Foot-and-mouth disease (FMD), caused by FMD virus (FMDV), is a contagious disease of even-toed ungulates, of which cattle, water buffalo, pigs, sheep and goats are the most important affected domesticated livestock species. Direct losses due to FMD infection include a drop in milk production, weight loss, reduced feed conversion and loss of draught power [1]. In addition, FMD free countries that experience an outbreak of FMD suffer high losses due to loss of access to export markets.

FMDV serotypes are based on complete absence of cross-protection. There are 7 serotypes of FMDV and many different genetic and antigenic variants within these serotypes which exhibit complete to partial degrees of cross-protection [2]. The level of cross-protection within a serotype can sometimes be low, and it is therefore essential to monitor whether new strains will escape vaccine-induced protection. In 2015, FMDV serotype A viruses from the A/ASIA/G-VII lineage (further denoted as G-VII), were detected in a number of countries in the Middle East. Sequence analysis revealed a close genetic relationship (>95% VP1 nucleotide identity) between viruses recovered from these outbreaks to FMD viruses circulating in South Asian countries, such as India and Bangladesh [3], and also demonstrated that these viruses were distinct (<84% VP1 nucleotide identity) from the A/ASIA/Iran-05 viruses that are endemic in the Middle East [4].

These outbreaks raised concern since initial *in vitro* laboratory analysis provided evidence for a poor antigenic match with vaccine strains used in the Middle East and vaccine strains available in vaccine banks of FMD free countries. In these vaccine banks, A_22_/IRQ/64, A/MAY/97 and A/IRN/05 are often available which share <85% VP1 sequence identity and represent distinct clades within the A/ASIA topotype. These vaccine strains also generated low geometric mean r_1_-values in the *in vitro* vaccine-matching test against representative G-VII isolates [5]. The calculated geometric mean r_1_-value, based on the data of Waters et al. [5], was 0.1 (95% Confidence Interval (CI) <0.04, 0.3>) for vaccine strain A_22_/IRQ/64; 0.2 (95% CI <0.1, 0.3>) for vaccine strain A/MAY/97 and 0.2 (95% CI <0.1, 0.4>) for another vaccine strain, A/SAU/95. The A/IRN/05 bovine vaccinal serum did not neutralise any of the G-VII isolates that were tested. These low r_1_-values (below 0.3) are generally considered indicative of a low antigenic match between the vaccine strain and field isolate, which may result in poor protection. However, emergency vaccines formulated with high antigen content from vaccine bank antigens often perform better than the results predicted from the *in vitro* vaccine matching test, and emergency vaccines with vaccine strains that have a relatively low r_1_-values against a field strain can provide sufficient intra-serotype heterologous protection [6,7,8,9]. In order to determine intra-serotype heterologous protection against G-VII, Waters et al. [5] performed an *in vivo* study to assess the level of protection provided by a single dose of a routinely used multi-valent vaccine containing A/IRN/05 and A/SAU/95 (as well as 3 serotype O antigens; O Manisa, O 3039, O PanAsia-2, and 1 serotype Asia1 antigen as well as 1 serotype SAT2 antigen). In their study, 9 of the 16 vaccinated cattle were protected after challenge with a representative G-VII virus. The mean neutralising antibody titres of the cattle against the challenge strains was 0.64–0.65 log_10_ lower than the mean titre against both serotype A vaccine strains. These results are indicative of an r_1_-value of 0.2. 

In view of the poor *in vitro* antigenic match of candidate serotype A vaccines and the suboptimal protection of tested vaccines [5], the objective of the current study was to quantify the level of protection provided in cattle by administration of monovalent emergency FMDV vaccines containing vaccine strains A_22_/IRQ/64 or A/MAY/97, formulated from vaccine bank antigen stocks, against challenge with a representative G-VII field isolate.

## 2. Materials and Methods

### 2.1. Vaccines

Two monovalent emergency vaccines, A_22_/IRQ/64 and A/MAY/97, were formulated from inactivated vaccine antigens that are held by the Australian Vaccine Bank. The vaccine was formulated as a double oil emulsion by Boehringer-Ingelheim, Pirbright and shipped to Wageningen Bioveterinary Research (WBVR) in Lelystad. Details about the adjuvant and antigen content were not provided by the producer. Both vaccines were administered intramuscularly in the neck.

### 2.2. Virus Strains and Cells

A representative field isolate (A/IRN/22/2015) from the G-VII lineage was obtained from the FAO World Reference Laboratory for FMD (WRLFMD) at Pirbright, UK, as an original suspension of the tongue epithelium collected in Iran. FMDV strains A_22_/IRQ/64 and A/MAY/97 that were used in the virus neutralisation test (VNT) were laboratory strains available at WBVR. Stock viruses for the VNT were grown in monolayers of IBRS-2 cells, titrated and stored at −70 °C until use. 

### 2.3. Reference Cattle Sera Collected from a Previous Experiment

Cattle sera collected 3 weeks after vaccination from a homologous A/MAY/97 potency test, performed according to the protocol outlined in the European Pharmacopeia using a commercial A/MAY/97 vaccine, were used for comparative analyses. In the homologous potency test, cattle were vaccinated with A/MAY/97 vaccine and after 21 days challenged with A/MAY/97 challenge virus. All 5 cattle receiving the full dose, and 4 out of 5 in both the 1/4 and 1/16 dose were protected, resulting in a potency of 18 PD_50_/dose (using the Spearman–Kärber method [10]).

### 2.4. Full Dose Protection Test Using A/MAY/97 and A_22_/IRQ/64 Vaccines

In the full dose protection test, monovalent emergency vaccine A_22_/IRQ/64 (group1) and A/MAY/97 (group2), were challenged with A/IRN/22/2015. All animal trials and laboratory work were performed at the high containment facilities of WBVR. The trials were performed in accordance to ethics approval from Australia (AEC1 1819) as well as the Netherlands (2016050.b). In the full dose protection test, a total of 17 mixed Dutch dairy breed cattle, 7–17 months old were selected from Dutch cattle herds. The cattle were randomly assigned to 3 groups, 2 groups of 7 cattle and 1 group of 3 unvaccinated control cattle. An unbalanced design with a lower number of control cattle was chosen on welfare grounds; power analysis showed that 7 vaccinated cattle and 3 controls were necessary (power = 0.8) to detect a one-sided significant difference (*p* = 0.05), if 60% of the vaccinated cattle were protected and none of the controls. The cattle were housed in a tie-stall at WBVR, and were fed grass pellets, oat-husk pellets and a limited amount of hay and had ad-lib drinking water; the acclimatisation period was 4 days. The first group of 7 cattle received a full dose of FMD monovalent emergency vaccine A_22_/IRQ/64, while the second group received a full dose of FMD monovalent emergency vaccine A/MAY/97. At 3 weeks post-vaccination, the vaccinated and control cattle were challenged by intradermolingual route with a 10^3.6^ PFU/mL of the original virus suspension of FMDV isolate A/IRN/22/2015 [5], injecting 0.1 mL at 2 sites.

After challenge, the cattle received Finadyne transdermal pour-on (MSD Animal Health) every 3 days, for analgesia using the dose (3.33 mg flunixin/kg bodyweight) prescribed by the producer. At the end of the study, 8 days post challenge (DPC), the cattle were euthanised using an overdose of sodium pentobarbital (Dechra Pharmaceuticals).

### 2.5. Heterologous (PD_50_) Potency Test with the A/MAY/97 Vaccine 

The potency test was performed using monovalent emergency vaccine A/MAY/97 (full dose, 1/3 dose, 1/9 dose) and challenge with A/IRN/22/2015, in accordance to ethics approval from Australia (AEC1 1859), the Netherlands (2016.D-0062.003) and the UK (Animal Welfare and Ethical Review Board AWERB of the Pirbright Institute). In total 18 cattle, mixed Dutch dairy breeds, 8–13 months old were selected from Dutch cattle herds. The cattle were randomly assigned to 4 groups, 3 groups of 5 vaccinated cattle and 1 group of 3 unvaccinated control cattle. Animal husbandry was the same as in the first trial with one exception that the cattle were housed free roaming, in 8 small stables, 12 cattle in pairs, and 6 cattle in groups of 3. The first group of 5 cattle received a full dose of FMD monovalent emergency vaccine containing vaccine strain A/MAY/97, the second group of 5 cattle a 1/3 dose and the third group of 5 cattle a 1/9 dose. At 3 weeks post-vaccination the cattle were challenged with FMDV isolate A/IRN/22/2015 obtained from the tongue of one of the control cattle in the previous experiment and diluted in minimal essential medium with Hanks’ salts supplemented with 2% foetal bovine serum and 2% antibiotic cocktail to a concentration of 10^5.4^ PFU/mL. The virus was injected intra-dermally at 2 sites, 0.1 mL per site, in the tongue. Analgesia and euthanasia were applied as was done in the full dose protection test. 

### 2.6. Clinical Observations and Sampling

Cattle were inspected for clinical disease daily and rectal temperatures were recorded. In addition to daily clinical inspections, the cattle were inspected under anaesthesia for presence of FMDV lesions at 4 DPC. The final reading of the test (lesion score) was at post-mortem 8 DPC. Serum samples were collected once (heterologous potency test) or twice (full dose protection test) per week before challenge and daily after challenge. Mouth swabs (Salivette^®^ tubes) and nose swabs were collected daily after challenge. All samples were directly processed in the laboratory and stored at −70 °C until testing.

### 2.7. Virus Isolation

Serum as well as nose and mouth swabs were tested for presence of FMDV using a plaque titration, by inoculating 200 µL of tenfold dilutions of the sample on two wells of primary lamb kidney cells grown in a collagen coated 6-well plate (Greiner^®^) as previously described [11]. Virus titres were expressed as log_10_ plaque forming units (pfu)/mL.

### 2.8. FMDV Genome Detection

Serum as well as nose and mouth swabs were tested for presence of FMDV genome using a real time RT-PCR [12]. RNA extraction was performed using the Magna Pure LC total Nucleic Acid Isolation kit (03 038 505) in the MagNaPure 96 system (Roche^®^, Mannheim, Germany). Extracted RNA was tested as described previously [12] using a LightCycler 480 Real-Time PCR System (Roche^®^).

### 2.9. Virus Neutralisation Tests

Neutralising antibody titres of all sera were determined against the following FMDV isolates: A/IRN/22/2015 (G-VII challenge virus), A/MAY/97 (homologous vaccine virus) and for samples of the full dose protection test also against A_22_/IRQ/64 (alternative homologous vaccine virus). The VNT was performed as described before [13] using IBRS-2 cells (CCLV-RIE 0103) [14] instead of BHK-21 cells. 

### 2.10. r_1_-Value Determination

For each individual cow in the heterologous potency test, the results of the 3 week post-vaccination sera were used to calculate the r_1_-value [15]. Subsequently, the r_1_-values were log transformed to calculate the geometric mean, standard deviation and 95% CI.

### 2.11. Antibodies Against Non-Structural Proteins

Antibodies against non-structural proteins were determined using the PrioCHECK™ FMDV NS Antibody ELISA Kit (ThermoFischer Scientific) as specified by the producer.

### 2.12. Statistical Analysis 

Differences in proportions of cattle protected against virulent challenge (e.g., difference in number of protected cattle) were tested with the Fischer’s exact test. The potency of the A/MAY/97 vaccine was calculated using both the method of Spearman–Kärber [10] and logistic regression. In the logistic regression protection was used as result variable and “0.94 × log_e_ (vaccine dose)” was used as offset. The value 0.94 is based on the common slope observed in 51 previously performed potency tests with FMDV oil adjuvanted vaccines (A. Dekker, personal observation). 

For the analysis of continuous data in multiple groups (e.g. duration of virus excretion in 4 groups), we used an ANOVA to test for statistical differences between groups. If a statistical difference was found, a pairwise *t*-test (with Holm correction) was used to analyse differences between groups. 

Sequential serological data (VNT) were analysed in a linear mixed effects model [16], with the neutralising antibody titre against A/IRN/22/2015 as result variable and the animal as random variable. The possible explanatory variables were DPC (as factor) and strain (full dose protection test) or dose (heterologous potency test) used for vaccination. 

The relation between neutralising antibody titre 3 weeks after vaccination and protection was analysed using logistic regression. The analysis included the sera of the homologous A/MAY/97 potency test previously performed and the sera of the A/MAY/97 vaccinated cattle challenged with A/IRN/22/2015 in either the full dose protection test or the heterologous potency test. The result variable was protection and the explanatory variables were the neutralising antibody titre against the homologous A/MAY/97 virus and whether or not the challenge was homologous. The significance of the homologous challenge was tested using a likelihood ratio test.

The relation between neutralising antibody titre 3 weeks after vaccination and vaccine dose, using the reference sera from the standard potency test as well as the sera from the heterologous potency test, were analysed using linear regression. In the analysis the neutralising antibody titre was the result variable, the logarithm of the vaccine dose, experiment and vaccine were possible explanatory variables. 

In all models, explanatory variables were selected based on the lowest AIC using forward selection. Statistical analysis was performed using R (version 3.3.1) [17].

## 3. Results

### 3.1. Protection and Virological Data for Full Dose Protection Test

The clinical outcome and virological data from the full dose protection test are summarised in Table 1. Only 2 of the 3 control cattle had clear FMD lesions on 2 or more feet. The third control cow had obvious FMD lesions in the mouth, but only superficial lesions were observed on 2 feet (indicated as doubtful (D) in Table 1). Two of the cattle vaccinated with A/MAY/97 (1234 and 1237) had a doubtful lesion at one of the feet. No samples were taken as previous studies have shown that no virus is usually isolated at 8 DPC. When the doubtful lesions in the vaccinated cattle were considered negative for FMDV infection, all the cattle vaccinated with A/MAY/97 were protected compared to only 2 of the 7 (29%) cattle vaccinated with A_22_/IRQ/64, resulting in a significant difference between A_22_/IRQ/64 and A/MAY/97 (*p* = 0.02, Fischer exact test). However, when the doubtful feet lesions were considered positive, 5 of the 7 (71%) A/MAY/97 cattle were protected and the difference between the 3 groups were not significant (*p* = 0.19, Fisher exact test). 

Viraemia, defined as isolation of infectious virus from serum, was not observed in any of the vaccinated cattle. In one of the cattle vaccinated with A_22_/IRQ/64, FMDV genome could be detected by RT-PCR for 1 day. Only control cattle developed viraemia, therefore statistical analysis of viraemia was not considered relevant. Live virus could be detected in mouth swabs of all cattle and the nose swabs of 16 out of 17 cattle, regardless of vaccination (Table 1). The duration of FMDV detection in nose swabs (*p* = 0.02) and mouth swabs (*p* < 0.01) was significantly different between groups (ANOVA). A significant difference between A/MAY/97 vaccinated cattle and the control group (*p* < 0.01 for mouth swabs, *p* = 0.02 for nose swabs) as well as between the A_22_/IRQ/64 vaccinated cattle and the control cattle (*p* < 0.01 for mouth swabs, *p* = 0.03 for nose swabs) was observed, but no difference between both groups of vaccinated cattle (pairwise *t*-test). 

The maximum virus titre observed in the A/MAY/97 and A_22_/IRQ/64 vaccinated cattle was significantly lower in nose swabs (ANOVA *p* < 0.01, *p* < 0.01 for A/MAY/97 versus controls, and *p* = 0.02 for A_22_/IRQ/64 versus controls) compared to the control cattle, but no difference was found between the vaccine groups (pairwise *t*-test). No difference in maximum virus titre in the mouth swabs was observed between the groups (ANOVA *p* = 0.07). 

Statistical analysis of genome detection produced in most cases the same result, but since genome detection is less relevant for transmission, the results are not reported.

### 3.2. Neutralising Antibody Titres in the Full Dose Protection Test

**Figure 1:**
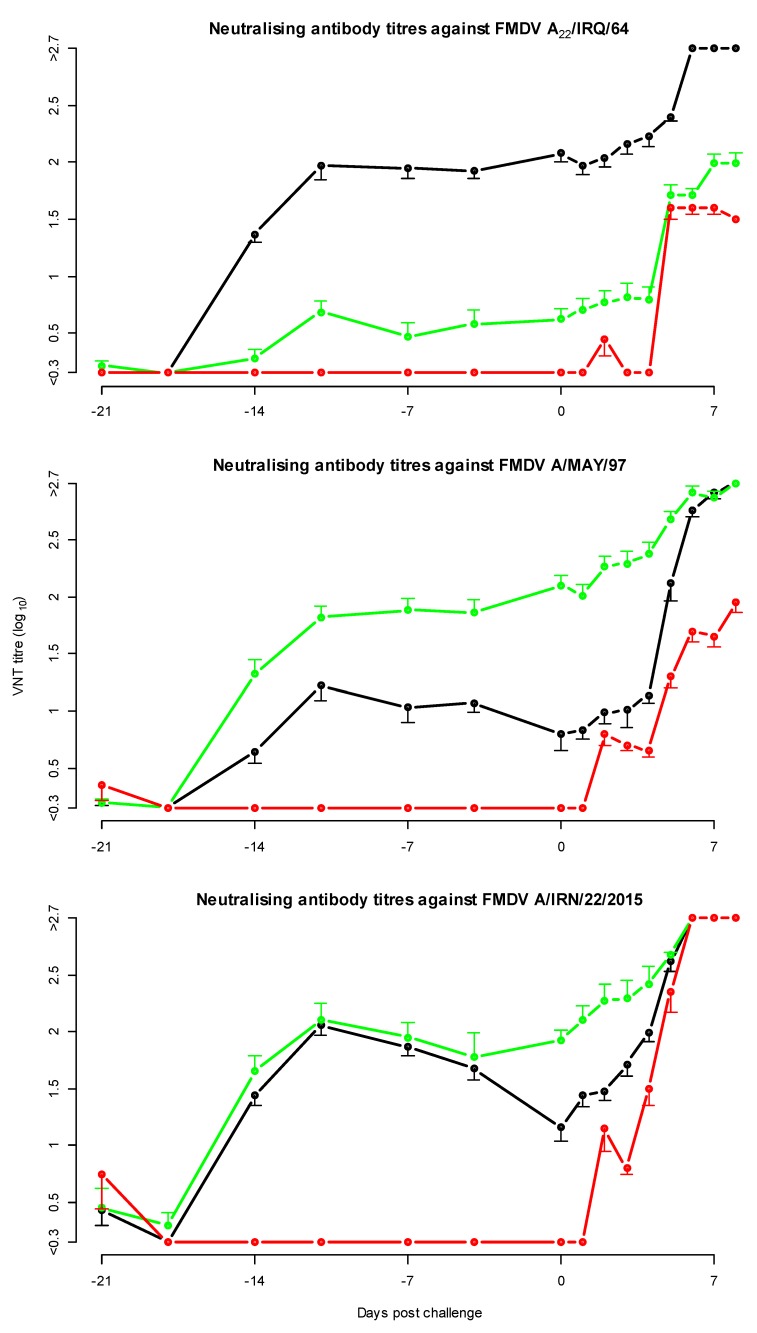
Mean neutralising antibody titres in the full dose protection test with A_22_/IRQ/64 vaccine virus and A/MAY/97 vaccine virus, and A/IRN/22/2015 challenge virus. The error bars, indicating the standard error of mean, are only shown on one side to avoid overlap. In black, the cattle vaccinated with A_22_/IRQ/64 vaccine, in green the cattle vaccinated with A/MAY/97 vaccine and in red the non-vaccinated control cattle.

All vaccinated cattle developed neutralising antibodies against the vaccine strain, as well as cross-neutralising antibodies against the G-VII strain A/IRN/22/2015 and in all groups an increase in antibody titres was observed post challenge (Figure 1). The neutralising antibody titres directed against the vaccine strain were generally higher than the neutralising antibody titres against the heterologous challenge virus (A/IRN/22/2015). Initially, both vaccines induced the same level of cross-neutralising antibodies measured using the A/IRN/22/2015 neutralisation test, but at the day of challenge the average heterologous antibody titre was 1.9 and 1.2 for the A/MAY/97 and the A_22_/IRQ/64 vaccinated groups respectively. At the same time point, the average neutralising antibody titres against the homologous vaccine strain for both the A/MAY/97 and A_22_/IRQ/64 vaccinated cattle were 2.1.

Using the sera collected at 3 weeks post-vaccination, the geometric mean r_1_-value for the A_22_/IRQ/64 vaccine against the challenge strain (A/IRN/22/2015) was 0.1 (95% CI <0.05, 0.3>) and for the A/MAY/97 vaccine it was 0.7 (95% CI <0.2, 2.2>). The geometric mean r_1_-value was high, and the CI was very wide because one of the sera of the A/MAY/97 had a 2-fold higher neutralising antibody titre against the challenge strain (A/IRN/22/2015), than against the homologous vaccine strain.

### 3.3. Protection and Virological Data for Heterologous Potency Test Using A/MAY/97 Vaccine

In the heterologous potency test, using the A/MAY/97 vaccine, all 5 cattle receiving a full vaccine dose, 4 out of 5 cattle receiving a 1/3 dose and 2 out of 5 cattle receiving a 1/9 dose were protected from FMD generalisation. Based on this result, a heterologous potency of 6.5 PD_50_/dose (95% CI <3, 13>) was calculated using the method of Spearman–Kärber [10]. Using logistic regression, with the slope of previous experiments as offset, a slightly higher potency with a higher upper limit of the 95% confidence interval was calculated (11 PD_50_/dose, 95% CI <3, 38>). 

The protection and virological data from this heterologous potency test are summarised in Table 2. Viraemia (live FMD virus detected in serum) was not detected in any vaccinated animals, but FMDV genome was detected in 4 of the vaccinated cattle (1 vaccinated with a 1/3 dose and 3 with 1/9 dose). In all cattle, FMD virus was detected in the nose and mouth swabs. The duration of virus detection was significantly different between the 4 groups in both serum (*p* ≪ 0.01) and nose swabs (*p* = 0.02). Also, the maximum amount of virus detected in serum (*p* ≪ 0.01) and nose swabs (*p* = 0.03), was different between groups. However, the duration of virus detection as well as the maximum amount of virus, in one or all vaccinated groups differed significantly from the control group (pairwise *t*-test), but there was no significant difference between the different dose groups. In the mouth samples, no significant differences were seen in the duration and maximum amount of virus detection between the groups (*p* = 0.3). Similar results were obtained when analysing FMDV genome detection; with only significant differences in duration and the Ct of genome detection as well as the minimal Ct (highest amount of genome) in serum and nose swabs between vaccinated and control cattle, but not between the different doses of vaccine applied.

### 3.4. Neutralising Antibody Titres in the Heterologous Potency Test

**Figure 2:**
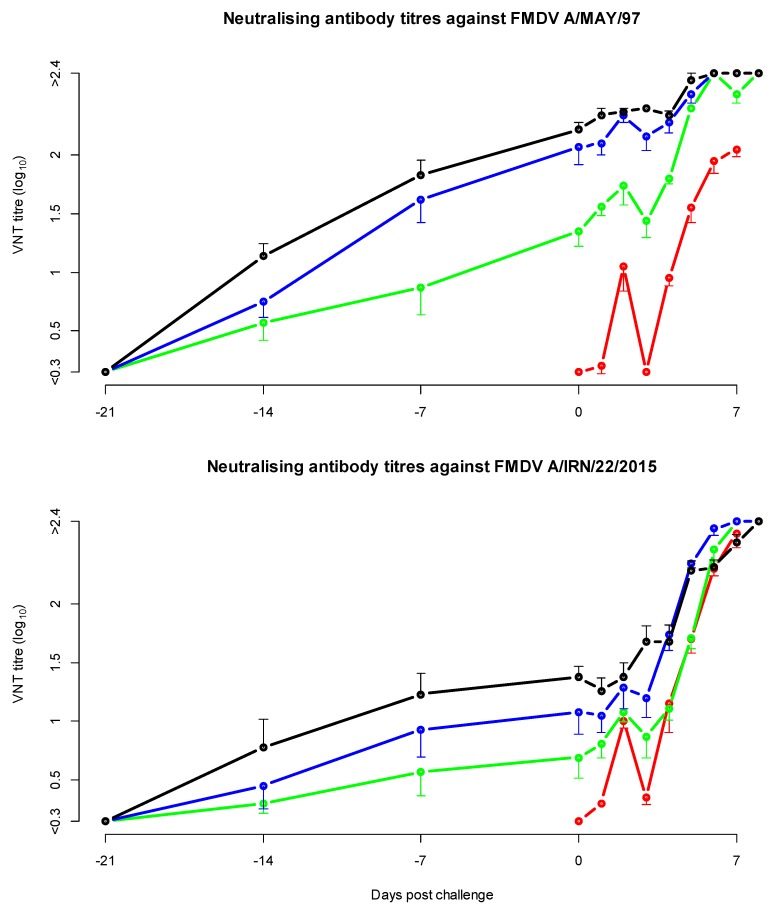
Mean neutralising antibody titres in the cattle potency test with A/MAY/97 vaccine and A/IRN/22/2015 challenge. The error bars, indicating the standard error of mean, are only shown on one side to avoid overlap. In black the cattle vaccinated with a full dose of vaccine, in blue the cattle vaccinated with a 1/3 dose, in green the cattle vaccinated with a 1/9 dose and in red the non-vaccinated control cattle.

Groups that received a higher vaccine dose developed significantly higher neutralising antibody titres (Figure 2 and Table 2), in both the homologous A/MAY/97 and heterologous A/IRN/22/2015 VNT (linear mixed effects model). In this model there was also a significant difference between DPC, and virus used in the neutralisation test, which can also be seen in Figure 2, the VNT titres increased after challenge and the homologous titres before challenge were higher than the heterologous titres. 

On the day of challenge, the neutralising antibody titre against the homologous vaccine strain A/MAY/97 was 0.3 log_10_ to 1.0 log_10_ higher compared to the titre against the challenge virus A/IRN/22/2015. Based on all 3-week post-vaccination sera, a geometric mean r_1_-value of 0.1 (95% CI <0.04, 0.6>) was calculated.

### 3.5. Antibodies Against Non-Structural Proteins

In both the full dose protection test and the heterologous potency test all cattle became positive for NS antibodies at a median time of 7 DPC (see Supplementary Tables).

### 3.6. Relationship between Neutralising Antibody Titres and Protection, for Estimation of Homologous Potency

**Figure 3:**
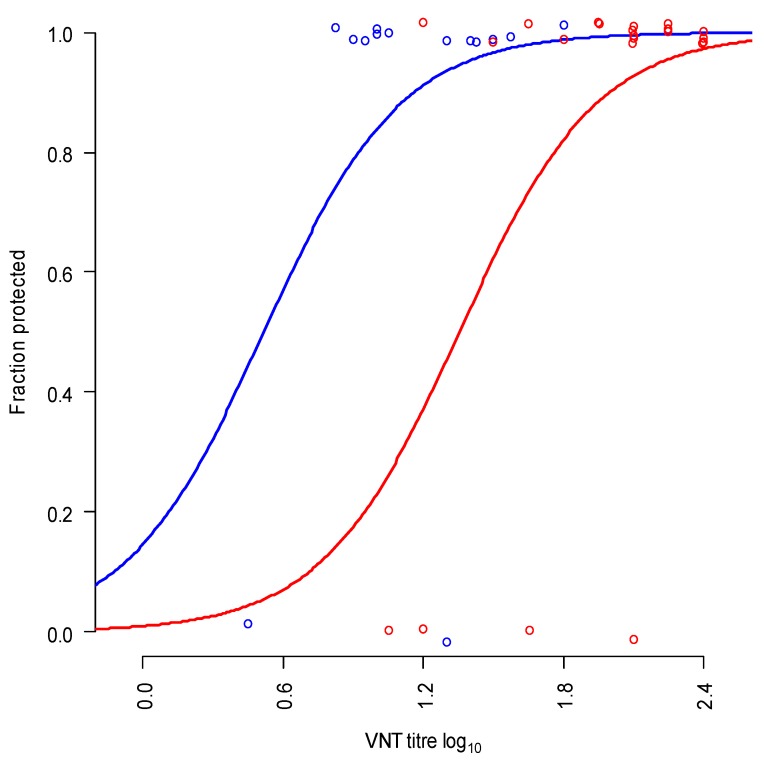
Relationship between neutralising antibody titre against A/MAY/97 and protection in both homologous (blue dots and line; using sera of a previous homologous potency test) vaccinated and heterologous vaccinated cattle (red dots and line; sera from both the full dose protection test and the heterologous potency test). The circles represent the observations in the experiments (0 = non-protected, 1 = protected, circles are slightly displaced to avoid overlap).

The reference sera of the homologous potency test with commercial A/MAY/97 vaccine showed a significant relationship between neutralising antibody titre and protection (Figure 3, blue dots and line). In the same analysis the sera of the full dose protection test as well as the heterologous potency test were analysed (Figure 3, red dots and line), the slope of the curves was not significantly different, but the position of the curves was different (*p* = 0.03, likelihood ratio test). The predicted neutralising antibody titre needed for 50% protection was 0.5 log_10_ for homologous (A/MAY/97) challenged cattle and 1.35 for heterologous (A/IRN/22/2015) challenged cattle. The neutralising antibody titres found in both the full dose protection test and the heterologous potency test (red dots Figure 3) correspond with a high level of homologous protection (Mean 97%, minimal 86%, maximum 100%). Due to the fact that there were no cattle with a low homologous protection it was not possible to estimate the homologous potency with high precision from the estimated homologous protection.

### 3.7. Relationship between Vaccine Dose and Neutralising Antibody Titres, for Estimation of Homologous Potency

**Figure 4:**
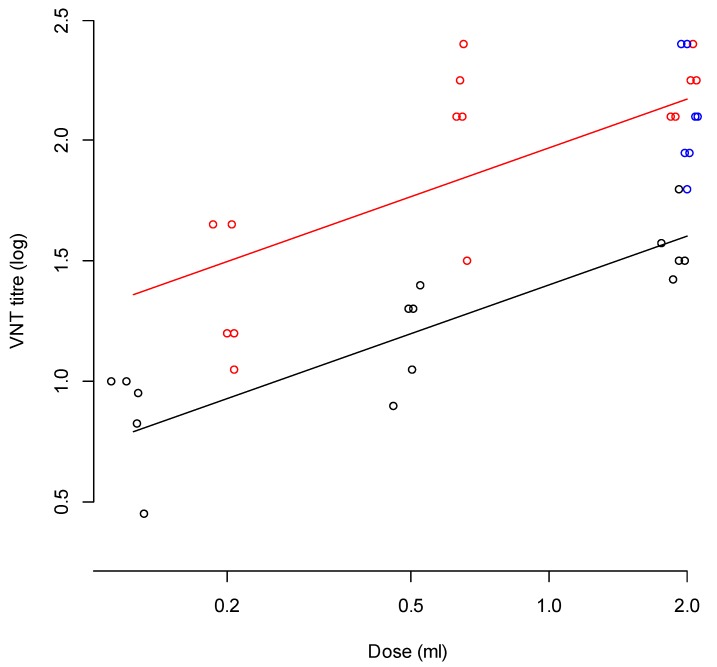
Relation between dose and the 3-week post-vaccination homologous neutralising antibody titres (including regression lines) against A/MAY/97 observed in a potency test with commercial A/MAY/97 vaccine (black), and the emergency A/MAY/97 vaccine (the full dose protection test in blue and the heterologous A/MAY/97 potency test in red). All data points are slightly displaced to avoid overlap. The lines represent the linear estimates of the relation between dose and neutralising antibody titre.

As the prediction of the homologous protection was not suitable for estimation of the homologous potency, we also analysed the relation between vaccine dose and neutralising antibody titre in both the reference sera of the homologous potency test as well as the sera from the full dose protection test and the heterologous potency test. Using linear regression with the VNT titre as result variable and the logarithm of the dose and type of vaccine (commercial A/MAY/97 vs. emergency A/MAY/97 vaccine) as explanatory variables. In Figure 4, the relationship between the vaccine dose and VNT titres are plotted (all 3 studies). In the full dose protection test, described in this paper, a full dose vaccine (2 mL) was used, in the heterologous protection study, 2 mL (full dose), 0.67 mL (1/3 dose) and 0.22 mL (1/9 dose) were used. In the homologous protection test that was performed previously, doses of 2 mL (full dose), 0.5 mL (1/4 dose), and 0.125 mL (1/16 dose) were used. The neutralising antibody titres of the heterologous studies using emergency vaccine were higher than the neutralising antibody responses using commercial vaccine (Figure 4). 

The homologous A/MAY/97 potency test (using commercial vaccine), resulted in a potency estimate of 18 PD_50_/dose (95% CI <9, 40>) when using Spearman–Kärber, or 44 PD_50_/dose (95% CI < 11, 179>) when calculating the potency with a logistic regression model in which the slope was fixed on the estimate from previous experiments [18] using “0.94 × log_e_(vaccine dose)” as offset. The average titre of the full dose of the commercial vaccine correlates with 1/7 dose of the emergency vaccine (Figure 4), suggesting a 7 times higher potency in the emergency vaccine compared to the commercial vaccine, leading to an estimate of the homologous potency of the emergency vaccine of 127 PD_50_/dose (based on Spearman–Kärber) (95% CI <39, 412>) or 303 PD_50_/dose (based on logistic regression with a common slope) (95% CI <27, 1606>). 

## 4. Discussion

The objective of the current study was to quantify protection provided by monovalent FMDV emergency vaccines A_22_/IRQ/64 or A/MAY/97 against challenge with a representative field isolate from the emerging G-VII lineage (A/IRN/22/2015). Our results showed that the FMDV A_22_/IRQ/64 emergency vaccine only protected 2 of 7 cattle against heterologous challenge, whereas the FMDV A/MAY/97 emergency vaccine had a higher level of protection. To further quantify the level of protection and possible in-vitro prediction of this protection, a full potency test with A/MAY/97 emergency vaccine was carried out. In both experiments the challenge dose was different, the titre of the original virus was relatively low, but to avoid the use of an extra cow for a cattle passage we collected vesicular material in the first experiment and used this in the second experiment. In the second experiment a dose was chosen that would be similar to 10,000 bovine ID_50_ [11]. Earlier studies have shown that the challenge dose not influence the outcome of a potency test [19], the difference in challenge dose is therefore not considered relevant. This experiment resulted in a heterologous potency of 6.5 PD_50_/dose (95% CI <3, 13>) when using the method of Spearman–Kärber, or 11 PD_50_/dose, 95% CI <3, 38>) when using logistic regression with the slope of previous experiments as offset. The World Organisation for Animal Health considers 3 PD_50_/dose (or 75% protection) against homologous challenge sufficient for FMDV vaccines [20], which would also be sufficient for heterologous challenge, indicating that the emergency A/MAY/97 vaccine, which are formulated to have a higher potency, should provide sufficient protection against the G-VII lineage viruses. From the results of our experiments we can calculate the cross-protection-ratio [21].

$$\mathrm{Cross-protection-ratio}=\;\frac{Heterologous\;potency}{Homologous\;potency}$$

Depending on the method used for calculation, this cross-protection-ratio was 6.5/127 = 0.05 (for the Spearman–Kärber estimates) or 11/303 = 0.04 (for the logistic regression estimates). Considering the wide confidence intervals of the calculated potencies, these result match well with the observed r_1_-value in the heterologous potency test (0.15, 95% CI <0.04, 0.6>), indicating that the r_1_-value might be a good predictor of the cross-protection-ratio, although an r_1_-value below 0.3 is often considered an indicator of poor protection. The formula above shows that one should not use an absolute cut-off for the cross-protection-ratio (and also not for the r_1_-value) but should always evaluate it in relation to the homologous potency. In many studies >6 PD_50_/dose against homologous challenge is considered sufficient for an emergency vaccine. The formula above shows that under the assumption that one should have at least 3 PD_50_/dose against the heterologous field virus; the cross-protection-ratio (and probably the r_1_-value) should not be below 0.5. However, the homologous potency of emergency vaccines is often much larger than 6 PD_50_/dose [6,7,8,9,22]. Therefore, managers of vaccine banks should not require vaccine of >6 PD_50_/dose but should know the homologous potency to be able to predict the protection against circulating field viruses, using in-vitro vaccine matching results (e.g., r_1_-value) as predictor of the cross-protection-ratio. Although in this study and a previous study [22] the cross-protection-ratio matches reasonably well with the r_1_-value, more research is needed to validate this for more strains, especially as the variation in both r_1_-value determination and potency tests is considerable [23,24,25,26].

Initially a geometric mean r_1_-value for FMDV A/MAY/97 vaccine, determined at the WRLFMD, was 0.2 (95% CI <0.1, 0.3>) [5]. In our full dose protection test, an r_1_-value of 0.7 (95% CI <0.2, 2.2>) was calculated. However, the 3-week post-vaccination sera collected in the heterologous potency test produced an r_1_-value of 0.15 (95% CI <0.04, 0.6>), which matches better with the previous WRL results. The reason behind the discrepancy of r_1_-values between the two experiments was not clear; all controls in the virus neutralisation tests were valid. These discrepancies were not followed up as the main objective was the determination of the potency after heterologous challenge, but these findings emphasise the inherent variability and difficulties when determining r_1_-values using VNT. Since r_1_-values are often used to select the vaccine strain for FMD control, the variation in r_1_-values between laboratories and between tests should be taken into account. The choice of the best vaccine strain should be made by considering also other information, e.g., the r_1_-value results of other genetically linked strains, and genetic relationships. 

In the full dose protection test dubious lesions were observed, which shows that it is not always easy to identify FMDV specific lesions. Virus isolation was not attempted, as many FMDV specific lesions in potency tests at 8 DPC are negative in virus isolation (A. Dekker, personal observation). The doubtful lesions were limited to one foot in each cow and the neutralising antibody titres against A/IRN/22/2015 in these two vaccinated cows were high. Both virus isolation and genome detection provided negative results in the vaccinated cows but were positive in the control cows. So, it is likely that these observed doubtful lesions in the vaccinated cattle were caused by something other than FMDV infection.

We statistically tested duration of virus detection, maximum virus titre, duration, and levels of genome detection in mouth, nose, and serum samples. These analyses focused on maximum virus titre in samples instead of the mean titre, under the assumption that the maximum titre is more deterministic than the mean titre as a factor that influences the probability of transmission. When using intra dermal injection in the tongue, in cattle that are protected one can still detect large amounts of FMDV in the mouth samples, because of the lesions induced in the tongue epithelium. In studies using more natural infection routes in which the epithelium is not damaged, the amount of virus in the mouth of contact infected cattle is much lower; in mouth swabs of contact exposed cattle the maximum titre was approximately 5.7 log_10_ PFU/mL [27] whereas in our experiment a maximum titre of 8.3 log_10_ PFU/mL were found. The local virus replication in the tongue is probably the reason that it was not possible to consistently detect significant differences between vaccinated and control cattle in the amount of virus detected in mouth swabs. Although needle challenge experiments are extremely valuable in quantification of vaccine-induced protection, the virus isolation results from mouth samples are less relevant for estimation the reduction of virus excretion. For this reason we also took nose samples, but the mean and maximum virus titre found in nose samples in our experiments (mean titre in positive nose samples was 1.7 log_10_ PFU/mL and maximum 4.7 log_10_ PFU/mL) was lower than the amount detected in mouth samples of contact infected cattle in previous experiments (see above), indicating that the excretion from the mouth after natural infection is probably more important for FMDV transmission between cattle. Especially because the amount of saliva that infected cattle excrete is higher than the amount of material that drips from the nose of an infected cow. Nevertheless, we could show a significant reduction of virus excretion from the nose in vaccinated cattle compared to non-vaccinated cattle, which is most likely the reason that transmission of FMDV between well vaccinated cattle is absent [27]. 

## 5. Conclusions

Our studies showed that FMDV emergency vaccine A/MAY/97 can protect against challenge with an FMDV field isolate belonging to the A/ASIA/G-VII lineage. Both the A/MAY/97 and A_22_/IRQ/64 vaccines shortened the duration of viraemia and excretions in nose and mouth swabs and decreased the maximum amount of virus detected. Although the A_22_/IRQ/64 vaccine did not provide full protection, it could still assist in controlling outbreaks. These findings indicate that it would be advisable to include the A/MAY/97 strain in European vaccine banks, although since this study has been completed, tailored A/ASIA/G-VII vaccines have become available from international suppliers, which might be another option to include.

## Figures and Tables

**Table 1:** Clinical, serological (virus neutralisation test (VNT) titre against challenge strain A/IRN/22/2015 at the day of challenge) and virological results in the 8-day period following challenge, in the full dose protection test in cattle vaccinated with A_22_/IRQ/64 and A/MAY/97 vaccine, and unvaccinated animals challenged with A/IRN/22/2015. For foot lesions a + indicates that foot lesions were present, a D indicates that a doubtful foot lesion was observed and a - indicates that no foot lesions were observed.

	Cow Number	Foot Lesions	VNT Titre at Day of Challenge ^1^	Duration Virus Detection (Days)			Maximum Titre (Log PFU/mL)			Duration Genome Detection (Days)		
				Serum ^2^	Nose ^3^	Mouth ^4^	Serum ^2^	Nose ^3^	Mouth ^4^	Serum ^2^	Nose ^3^	Mouth ^4^
Vaccine A_22_/IRQ/64	1227	-	1.2	0	0	6	0.0	0.0	6.1	2	5	6
	1228	+	1.05	0	2	5	0.0	2.6	6.8	3	8	8
	1229	+	1.5	0	2	6	0.0	2.1	7.4	2	7	8
	1230	+	1.5	0	2	4	0.0	1.2	5.5	1	7	8
	1231	+	1.05	0	5	6	0.0	3.3	5.2	2	8	8
	1232	-	1.2	0	2	6	0.0	1.7	7.9	2	7	7
	1233	+	0.6	0	4	5	0.0	2.6	6.7	4	7	8
Vaccine A/MAY/97	1234	D	1.95	0	2	5	0.0	1.2	4.4	0	6	7
	1235	-	1.95	0	3	6	0.0	1.4	5.9	0	6	8
	1236	-	2.1	0	1	6	0.0	0.4	4.9	0	6	7
	1237	D	2.1	0	1	6	0.0	0.4	6.6	2	7	8
	1238	-	2.1	0	1	5	0.0	0.4	4.5	0	3	5
	1239	-	1.5	0	2	6	0.0	1.7	6.7	0	6	8
	1240	-	1.8	0	4	5	0.0	1.8	7.5	0	5	8
Control	1241	D	<0.6	3	4	8	3.3	3.5	4.5	5	8	8
	1242	+	<0.6	3	6	8	3.4	3.7	5.0	4	8	8
	1243	+	<0.6	3	5	8	3.8	3.8	4.7	5	7	8

^1^ VNT titres at 0 DPC from supplement Table S1c, ^2^ Serum data from supplement Table S2, ^3^ Nose swab data from supplement Table S3, ^4^ Mouth swab data from supplement Table S4.

**Table 2:** Clinical, serological (VNT titre against challenge strain A/IRN/22/2015 at the day of challenge) and virological results in the 8-day period following challenge, in the potency test using A/MAY/97 vaccine. For foot lesions a + indicates that foot lesions were present, a - indicates that no foot lesions were observed.

	Cow Number	Foot Lesions	VNT Titre at Day of Challenge ^1^	Duration Virus Detection (Days)			Maximum Titre (log PFU/mL)			Duration Genome Detection (Days)		
				Serum ^2^	Nose ^3^	Mouth ^4^	Serum ^6^	Nose ^7^	Mouth ^8^	Serum ^6^	Nose ^7^	Mouth ^8^
Full dose	1566	-	1.2	0	5	6	0.0	2.7	7.7	1	6	8
	1567	-	1.5	0	3	7	0.0	1.1	7.4	1	7	7
	1568	-	1.2	0	3	4	0.0	1.9	5.2	0	6	7
	1569	-	1.65	0	1	6	0.0	0.7	8.3	1	3	8
	1570	-	1.35	0	1	5	0.0	0.9	7.6	2	4	7
1/3 dose	1571	-	1.35	0	2	5	0.0	0.9	8.0	1	7	8
	1572	-	1.05	0	2	5	0.0	2.6	7.9	2	7	8
	1573	-	1.65	0	0	5	0.0	0.0	5.0	0	3	6
	1574	-	0.75	0	2	5	0.0	3.3	7.1	1	5	6
	1575	+	0.6	0	4	7	0.0	1.0	7.1	1	8	8
1/9 dose	1576	+	0.9	0	2	6	0.0	0.9	6.2	4	6	8
	1577	-	1.2	0	1	7	0.0	0.4	7.5	2	4	8
	1578	-	0.75	0	3	5	0.0	1.9	6.4	0	4	8
	1579	+	0.3	0	5	5	0.0	3.2	7.0	4	7	8
	1580	+	0.3	0	3	6	0.0	3.6	7.4	4	5	8
Control	1581 *	+	<0.3	3	6	7	4.8	4.7	6.7	4	7	7
	1582	+	<0.3	3	6	6	3.9	3.9	7.1	5	8	8
	1583	+	<0.3	3	5	7	4.7	3.7	4.9	4	6	8

* Cow 1581 died 7 days post challenge. A high concentration of FMD genome was detected in the cardiac muscle and histology revealed infiltration of mononuclear cells and disintegration of muscle tissue. ^1^ VNT titres at 0 DPC from supplement Table S5b, ^2^ Serum data from supplement Table S6, ^3^ Nose swab data from supplement Table S7, ^4^ Mouth swab data from supplement Table S8.

## Supplementary Materials

The raw data are available online at https://www.mdpi.com/2076-393X/8/1/24/s1: Supplementary Table S1a. VNT titres against A_22_/IRQ/64 vaccine strain in vaccinated and unvaccinated cattle in the full dose protection test. Supplementary Table S1b. VNT titres against A/MAY/97 vaccine strain in vaccinated and unvaccinated cattle in the full dose protection test. Supplementary Table S1c. VNT titres against A/IRN/22/2015 challenge strain in vaccinated and unvaccinated cattle in the full dose protection test. Supplementary Table S2. Results of virus isolation and titration (VI; log_10_ PFU/mL) and viral RNA detected by real-time PCR (PCR; Cp values) from serum of vaccinated and unvaccinated cattle in in the full dose protection test with A_22_/IRQ/64 and A/MAY/97 vaccines (D = Close to detection limit of the PCR). Supplementary Table S3. Results of virus isolation and titration (VI; log_10_ PFU/mL) and viral RNA detected by real-time PCR (PCR; Cp values) from mouth swabs of vaccinated and unvaccinated cattle in the full dose protection test with A_22_/IRQ/64 and A/MAY/97 vaccines (D = Close to detection limit of the PCR. Supplementary Table S4. Results of virus isolation and titration (VI; log_10_ PFU/mL) and viral RNA detected by real-time PCR (PCR; Cp values) from mouth swabs of vaccinated and unvaccinated cattle in the full dose protection test with A_22_/IRQ/64 and A/MAY/97 vaccines (D = Close to detection limit of the PCR). Supplementary Table S5a. VNT titres against A/MAY/97 vaccine strain in vaccinated and unvaccinated cattle in the heterologous potency test. Supplementary Table S5b. VNT titres against A/IRN/22/2015 in vaccinated and unvaccinated cattle in the heterologous potency test. Supplementary Table S6. Results of virus isolation and titration (VI; log_10_ PFU/mL) and viral RNA detected by real-time PCR (PCR; Cp values) from serum of vaccinated and unvaccinated cattle in the heterologous potency test. Supplementary Table S7. Results of virus isolation and titration (VI; log_10_ PFU/mL) and viral RNA detected by real-time PCR (PCR; Cp values) from nose swabs of vaccinated and unvaccinated cattle in the heterologous potency test. Supplementary Table S8. Results of virus isolation and titration (VI; log10 PFU/mL) and viral RNA detected by real-time PCR (PCR; Cp values) from mouth swabs of vaccinated and unvaccinated cattle in the heterologous potency test. Supplementary Table S9a. Percentage inhibition in the Priocheck NS ELISA in the full dose protection test. Supplementary Table S9b. Percentage inhibition in the Priocheck NS ELISA in the heterologous protection test.
