# Supplementary material for: Cross-Protection Induced by a A/MAY/97 Emergency Vaccine Against Intra-Serotype Heterologous Challenge with a Foot-and-Mouth Disease Virus from the A/ASIA/G-VII Lineage
Source: Vaccines (Basel). 2020 Jan 14;8(1):24. doi: 10.3390/vaccines8010024 (PMC7157754; doi:10.3390/vaccines8010024)
Supplement: Supplementary file 1 [file vaccines-08-00024-s001.pdf]

Supplementary material

Supplementary Table 1a. VNT titres against A_{22}/IRQ/64 vaccine strain in vaccinated and unvaccinated cattle in the full dose protection test (animal numbers in bold indicate cattle with clear or doubtful foot lesions).

**Groups**	**Animal**	**Days post challenge**														
		**-21**	**-18**	**-14**	**-11**	**-7**	**-4**	**0**	**1**	**2**	**3**	**4**	**5**	6	7	8
A_{22}/IRQ/64	1227	<0.3	<0.3	1.20	1.80	2.10	2.10	2.10	2.10	2.10	2.25	2.40	2.40	>2.7	>2.7	>2.7
	**1228**	<0.3	<0.3	1.65	2.40	2.10	2.10	2.10	2.10	2.10	2.25	2.40	2.40	>2.7	>2.7	>2.7
	**1229**	<0.3	<0.3	1.35	2.10	1.95	1.95	2.25	2.10	2.25	2.40	2.40	2.40	>2.7	>2.7	>2.7
	**1230**	<0.3	<0.3	1.05	2.10	2.10	1.80	2.10	1.95	2.10	2.25	2.40	2.55	>2.7	>2.7	>2.7
	**1231**	<0.3	<0.3	1.50	1.95	2.10	1.80	2.10	1.95	1.95	2.25	2.10	2.25	>2.7	>2.7	>2.7
	1232	<0.3	<0.3	1.50	2.10	1.80	2.10	2.25	2.10	2.10	2.10	2.10	2.40	>2.7	>2.7	>2.7
	**1233**	<0.3	<0.3	1.35	1.35	1.50	1.65	1.65	1.50	1.65	1.65	1.80	2.40	>2.7	>2.7	>2.7
A/MAY/97	**1234**	<0.3	<0.3	<0.6	<0.6	<0.6	0.60	0.60	0.75	0.90	0.90	1.05	1.80	1.50	1.95	1.80
	1235	<0.3	<0.3	0.60	0.90	0.90	0.90	0.60	0.90	0.90	0.90	0.90	2.10	1.95	2.10	2.25
	1236	0.45	<0.3	<0.6	0.75	<0.6	<0.6	<0.6	<0.6	<0.6	<0.6	<0.6	1.50	1.80	2.40	2.40
	**1237**	<0.3	<0.3	<0.6	0.75	<0.6	<0.6	0.60	0.75	0.75	0.75	0.90	1.80	1.65	1.80	1.80
	1238	0.30	<0.3	<0.6	0.75	0.75	1.05	0.90	0.90	0.90	0.90	0.90	1.80	1.65	1.95	1.80
	1239	<0.3	<0.3	0.60	0.90	0.60	0.60	0.60	0.60	0.90	0.90	0.75	1.50	1.65	1.80	2.10
	1240	<0.3	<0.3	<0.6	0.60	0.60	0.60	0.90	0.90	0.90	1.20	0.90	1.50	1.80	1.95	1.80
Unvaccinated Controls	**1241**							<0.6	<0.6	0.60	<0.6	<0.6	1.80	1.65	1.65	1.50
	**1242**							<0.6	<0.6	<0.6	<0.6	<0.6	1.50	1.50	1.65	1.50
	**1243**							<0.6	<0.6	0.60	<0.6	<0.6	1.50	1.65	1.50	1.50

Supplementary Table 1b. VNT titres against A/MAY/97 vaccine strain in vaccinated and unvaccinated cattle in the full dose protection test

Groups	Animal	Days post challenge														
		-21	-18	-14	-11	-7	-4	0	1	2	3	4	5	6	7	8
A_{22}/IRQ/64	1227	<0.3	<0.3	0.60	0.75	0.60	1.20	0.75	0.90	1.20	1.05	1.20	2.25	2.70	>2.7	>2.7
	**1228**	<0.3	<0.3	1.05	1.35	1.20	0.75	0.90	0.75	1.20	1.20	1.20	2.40	>2.7	>2.7	>2.7
	**1229**	0.30	<0.3	<0.6	1.50	1.20	1.35	1.35	1.20	1.20	1.35	1.20	2.55	2.55	>2.7	>2.7
	**1230**	0.30	<0.3	0.60	1.05	0.75	0.90	0.60	0.60	0.60	1.05	1.35	2.55	>2.7	>2.7	>2.7
	**1231**	<0.3	<0.3	0.60	1.80	1.50	1.20	1.05	0.90	1.05	1.20	1.05	1.65	2.70	>2.7	>2.7
	1232	<0.3	<0.3	0.90	1.05	1.20	1.20	0.75	0.90	1.05	1.05	1.20	1.65	2.70	2.70	>2.7
	**1233**	<0.3	<0.3	0.60	1.05	0.75	0.90	<0.6	0.60	0.60	<0.6	0.75	1.80	2.70	2.70	>2.7
A/MAY/97	**1234**	<0.3	<0.3	1.80	1.80	1.80	1.80	2.10	1.80	2.25	1.95	2.40	2.55	>2.7	2.70	>2.7
	1235	<0.3	<0.3	1.80	2.25	2.25	2.40	2.40	2.40	2.40	2.55	2.55	2.70	>2.7	>2.7	>2.7
	1236	0.30	<0.3	1.05	1.50	1.65	1.65	1.80	1.80	1.80	1.80	1.80	2.40	>2.7	>2.7	>2.7
	**1237**	<0.3	<0.3	1.05	1.65	1.50	1.50	2.10	2.10	2.40	2.40	2.40	2.70	>2.7	2.70	>2.7
	1238	0.30	<0.3	1.20	2.10	2.10	1.95	2.40	2.25	2.40	2.40	2.55	2.70	>2.7	>2.7	>2.7
	1239	<0.3	<0.3	1.20	1.65	1.80	1.80	1.95	1.80	2.40	2.55	2.40	2.70	2.70	2.70	>2.7
	1240	<0.3	<0.3	1.20	1.80	2.10	1.95	1.95	1.95	2.25	2.40	2.55	>2.7	2.70	>2.7	>2.7
Unvaccinated Controls	**1241**							<0.6	<0.6	0.90	0.60	0.75	1.50	1.80	1.80	1.95
	**1242**							<0.6	<0.6	0.60	0.75	0.60	1.20	1.80	1.50	2.10
	**1243**							<0.6	<0.6	0.90	0.75	0.60	1.20	1.50	1.65	1.80

Supplementary Table 1c. VNT titres against A/IRN/22/2015 challenge strain in vaccinated and unvaccinated cattle in the full dose protection test (animal numbers in bold indicate cattle with clear or doubtful foot lesions).

Groups	Animal	Days post challenge														
		-21	-18	-14	-11	-7	-4	0	1	2	3	4	5	6	7	8
A_{22}/IRQ/64	1227	0.75	<0.3	1.35	1.95	1.95	1.80	1.20	1.50	1.65	2.10	1.95	2.70	>2.7	>2.7	>2.7
	**1228**	<0.3	<0.3	1.65	2.25	1.95	1.80	1.05	1.50	1.65	1.65	2.10	2.70	>2.7	>2.7	>2.7
	**1229**	0.75	<0.3	1.35	1.95	1.65	1.50	1.50	1.65	1.50	1.95	2.10	2.70	>2.7	>2.7	>2.7
	**1230**	0.90	<0.3	1.20	2.10	1.95	1.65	1.50	1.35	1.50	1.50	2.10	2.70	>2.7	>2.7	>2.7
	**1231**	<0.3	<0.3	1.80	2.40	1.95	1.95	1.05	1.50	1.35	1.50	1.65	2.10	>2.7	>2.7	>2.7
	1232	<0.3	<0.3	1.50	2.10	2.10	1.80	1.20	1.65	1.65	1.95	2.25	2.70	>2.7	>2.7	>2.7
	**1233**	<0.3	<0.3	1.20	1.65	1.50	1.20	0.60	0.90	1.05	1.35	1.80	2.70	>2.7	>2.7	>2.7
A/MAY/97	**1234**	<0.3	0.90	2.40	2.10	1.95	1.80	1.95	2.25	2.40	2.55	2.70	2.55	>2.7	>2.7	>2.7
	1235	<0.3	<0.3	1.80	2.70	2.40	2.10	1.95	2.40	2.10	2.25	2.40	2.70	>2.7	>2.7	>2.7
	1236	1.20	<0.3	1.50	2.25	2.25	2.70	2.10	2.40	2.55	2.40	2.70	2.70	>2.7	>2.7	>2.7
	**1237**	<0.3	<0.3	1.35	1.50	1.50	1.20	2.10	2.10	2.70	2.70	2.70	2.70	>2.7	>2.7	>2.7
	1238	1.05	<0.3	1.35	2.25	2.10	2.10	2.10	2.25	2.55	2.70	2.70	2.70	>2.7	>2.7	>2.7
	1239	0.30	0.45	1.50	1.65	1.50	1.20	1.50	1.50	1.65	1.65	1.65	2.70	>2.7	>2.7	>2.7
	1240	<0.3	<0.3	1.65	2.25	1.95	1.35	1.80	1.80	1.95	1.80	2.10	2.70	>2.7	>2.7	>2.7
Unvaccinated Controls	**1241**							<0.6	<0.6	1.35	0.90	1.65	2.70	>2.7	>2.7	>2.7
	**1242**							<0.6	<0.6	0.75	0.75	1.20	2.25	>2.7	>2.7	>2.7
	**1243**							<0.6	<0.6	1.35	0.75	1.65	2.10	>2.7	>2.7	>2.7

Supplementary Table 2. Results of virus isolation and titration (VI; log_{10} PFU/ml) and viral RNA detected by real-time PCR (PCR; Cp values) from serum of vaccinated and unvaccinated cattle in in the full dose protection test with A_{22}/IRQ/64 and A/MAY/97 vaccines (animal numbers in bold indicate cattle with clear or doubtful foot lesions).

Groups	Animal		0 DPC	1 DPC	2 DPC	3 DPC	4 DPC	5 DPC	6 DPC	7 DPC	8 DPC
A_{22}/IRQ/64	1227	PCR	-	33.08	31.67	-	-	-	-	-	-
		VI	-	-	-	-	-	-	-	-	-
	**1228**	PCR	-	32.68	31.87	32.16	-	-	-	-	-
		VI	-	-	-	-	-	-	-	-	-
	**1229**	PCR	-	-	-	32.49	31.57	-	-	-	-
		VI	-	-	-	-	-	-	-	-	-
	**1230**	PCR	-	32.73	-	-	-	-	-	-	-
		VI	-	-	-	-	-	-	-	-	-
	**1231**	PCR	-	-	32.23	-	31.77	-	-	-	-
		VI	-	-	-	-	-	-	-	-	-
	1232	PCR	-	32.76	32.08	-	-	-	-	-	-
		VI	-	-	-	-	-	-	-	-	-
	**1233**	PCR	-	31.50	31.98	31.11	31.68	-	-	-	-
		VI	-	-	-	-	-	-	-	-	-
A/MAY/97	**1234**	PCR	-	-	-	-	-	-	-	-	-
		VI	-	-	-	-	-	-	-	-	-
	1235	PCR	-	-	-	-	-	-	-	-	-
		VI	-	-	-	-	-	-	-	-	-
	1236	PCR	-	-	-	-	-	-	-	-	-
		VI	-	-	-	-	-	-	-	-	-
	**1237**	PCR	-	-	32.14	-	31.78	-	-	-	-
		VI	-	-	-	-	-	-	-	-	-
	1238	PCR	-	-	-	-	-	-	-	-	-
		VI	-	-	-	-	-	-	-	-	-
	1239	PCR	-	-	-	-	-	-	-	-	-
		VI	-	-	-	-	-	-	-	-	-
	1240	PCR	-	-	-	-	-	-	-	-	-
		VI	-	-	-	-	-	-	-	-	-
Unvaccinated Controls	**1241**	PCR	-	21.92	20.71	22.64	29.54	31.73	-	-	-
		VI		3.33	3.31	2.74	-	-	-	-	-
	**1242**	PCR	-	24.21	18.95	19.38	24.66	-	-	-	-
		VI	-	2.60	3.41	3.03	-	-	-	-	-
	**1243**	PCR	-	20.01	19.50	20.90	29.88	31.80	-	-	-
		VI	-	3.77	2.94	2.61	-	-	-	-	-

Supplementary Table 3. Results of virus isolation and titration (VI; log_{10} PFU/ml) and viral RNA detected by real-time PCR (PCR; Cp values) from nose swabs of vaccinated and unvaccinated cattle in the full dose protection test with A_{22}/IRQ/64 and A/MAY/97 vaccines (animal numbers in bold indicate cattle with clear or doubtful foot lesions).

Groups	Animal		0 DPC	1 DPC	2 DPC	3 DPC	4 DPC	5 DPC	6 DPC	7 DPC	8 DPC
A_{22}/IRQ/64	1227	PCR	-	-	28.84	27.66	32.13	31.94	31.69	-	-
		VI	-	-	-	-	-	-	-	-	-
	**1228**	PCR	-	25.63	29.66	25.72	31.09	30.99	32.67	32.46	31.83
		VI	-	2.64	1.90	-	-	-	-	-	-
	**1229**	PCR	-	31.97	24.93	30.46	31.63	31.74	31.67	-	31.74
		VI	-	-	2.14	1.18	-	-	-	-	-
	**1230**	PCR	-	-	29.80	29.90	30.32	30.17	31.70	32.57	32.42
		VI	-	-	1.24	0.70	-	-	-	-	-
	**1231**	PCR	-	31.71	21.57	28.16	26.91	29.27	29.91	28.23	31.48
		VI	-	1.00	3.25	2.18	2.49	1.00	-	-	-
	1232	PCR	-	32.25	29.46	29.90	28.29	30.56	31.83	33.43	-
		VI	-	-	0.88	-	1.70	-	-	-	-
	**1233**	PCR	-	31.92	29.11	26.99	23.43	18.87	30.73	18.70	-
		VI	-	1.00	2.42	-	2.61	1.88	-	-	-
A/MAY/97	**1234**	PCR	-	31.99	28.05	31.25	29.23	29.76	31.82	-	-
		VI	-	-	1.24	-	1.10	-	-	-	-
	1235	PCR	-	32.80	29.61	30.65	30.60	30.71	32.11	-	-
		VI	-	-	1.44	1.35	0.40	-	-	-	-
	1236	PCR	-	31.78	30.54	31.81	31.69	33.05	32.01	-	-
		VI	-	-	-	-	0.40	-	-	-	-
	**1237**	PCR	-	32.09	31.07	30.26	31.75	32.10	32.23	31.82	-
		VI	-	-	0.40	-	-	-	-	-	-
	1238	PCR	-	-	31.00	-	32.14	32.20	-	-	-
		VI	-	-	0.40	-	-	-	-	-	-
	1239	PCR	-	-	31.32	31.59	30.00	31.75	31.71	28.56	-
		VI	-	-	0.70	-	1.65	-	-	-	-
	1240	PCR	-	-	30.45	31.25	29.88	28.29	30.11	-	-
		VI	-	-	0.88	0.40	0.88	1.76	-	-	-
Unvaccinated Controls	**1241**	PCR	-	31.72	21.59	18.20	24.07	18.07	31.07	32.62	30.84
		VI	-	-	3.45	2.80	2.99	3.05	-	-	-
	**1242**	PCR	-	30.43	20.45	24.68	24.30	25.10	30.23	30.18	31.82
		VI	-	1.54	3.74	2.55	2.60	2.12	1.00	-	-
	**1243**	PCR	-	-	20.89	24.76	17.72	19.71	26.93	27.44	31.70
		VI	-	-	3.79	2.23	3.35	3.10	1.44	-	-

Supplementary Table 4. Results of virus isolation and titration (VI; log_{10} PFU/ml) and viral RNA detected by real-time PCR (PCR; Cp values) from mouth swabs of vaccinated and unvaccinated cattle in the full dose protection test with A_{22}/IRQ/64 and A/MAY/97 vaccines (animal numbers in bold indicate cattle with clear or doubtful foot lesions).

Groups	Animal		0 DPC	1 DPC	2 DPC	3 DPC	4 DPC	5 DPC	6 DPC	7 DPC	8 DPC
A_{22}/IRQ/64	1227	PCR	-	14.04	14.86	14.67	20.69	16.85	24.16	-	-
		VI	-	6.13	5.00	3.70	2.61	3.96	1.63	-	-
	**1228**	PCR	-	10.57	10.83	16.74	19.56	19.89	28.89	30.20	26.39
		VI	-	6.79	6.22	3.86	3.36	2.30	-	-	-
	**1229**	PCR	-	9.34	16.56	18.27	15.90	16.01	18.16	23.04	27.44
		VI	-	7.44	3.44	3.54	4.48	3.55	2.00	-	-
	**1230**	PCR	-	31.84	10.50	15.13	23.72	21.02	20.53	27.46	27.96
		VI	-	-	5.49	4.00	2.10	1.68	-	-	-
	**1231**	PCR	-	21.82	15.61	17.66	17.06	16.12	18.76	25.83	29.09
		VI	-	5.23	5.22	4.40	4.50	4.13	4.23	-	-
	1232	PCR	-	13.52	8.15	23.08	14.23	11.87	22.56	30.86	-
		VI	-	6.56	7.93	4.10	5.48	5.14	1.57	-	-
	**1233**	PCR	-	8.86	14.96	16.67	18.90	23.56	27.71	27.29	31.82
		VI	-	6.72	4.64	3.88	2.96	2.01	-	-	-
A/MAY/97	**1234**	PCR	-	24.45	17.69	20.71	28.42	29.18	31.80	31.74	-
		VI	-	2.78	4.44	4.10	2.06	1.51	-	-	-
	1235	PCR	-	30.63	11.64	11.90	16.90	15.62	18.58	29.63	31.77
		VI	-	1.24	5.90	5.83	4.48	3.97	2.24	-	-
	1236	PCR	-	27.14	16.49	23.53	17.05	28.56	30.52	32.14	-
		VI	-	2.64	4.47	1.30	4.90	0.40	0.70	-	-
	**1237**	PCR	-	25.81	9.77	17.79	13.00	17.98	19.53	24.67	31.73
		VI	-	2.68	6.60	4.35	6.06	3.07	2.28	-	-
	1238	PCR	-	25.68	17.51	27.59	30.86	29.96	-	-	-
		VI	-	2.48	4.53	1.48	1.22	1.00	-	-	-
	1239	PCR	-	20.92	10.87	17.66	20.79	22.72	27.62	29.56	30.68
		VI	-	3.25	6.65	4.18	2.00	2.57	1.78	-	-
	1240	PCR	-	11.46	8.62	19.92	20.02	15.16	22.44	27.63	31.83
		VI	-	6.47	7.53	3.13	3.54	4.66	-	-	-
Unvaccinated Controls	**1241**	PCR	-	30.52	15.83	19.61	19.01	18.58	22.47	21.49	22.60
		VI	-	2.71	4.53	3.35	2.80	3.04	2.67	2.08	1.51
	**1242**	PCR	-	23.47	12.41	15.96	20.21	14.03	25.30	24.70	21.66
		VI	-	2.89	5.04	2.92	3.40	4.61	2.12	1.54	0.88
	**1243**	PCR	-	26.80	12.59	17.33	18.08	18.09	18.71	18.93	20.64
		VI	-	2.60	4.68	2.66	3.43	3.51	3.60	1.40	0.88

Supplementary Table 5a. VNT titres against A/MAY/97 vaccine strain in vaccinated and unvaccinated cattle in the heterologous potency test (animal numbers in bold indicate cattle with foot lesions).

**Groups**	**Animal**	**Days post challenge**											
		-21	-14	-7	0	1	2	3	4	5	6	7	8
Full Dose (2ml)	1566	<0.3	1.20	1.65	2.10	2.10	2.25	2.40	2.25	>2.4	>2.4	>2.4	>2.4
	1567	<0.3	1.05	2.25	2.25	2.40	2.40	2.40	2.40	>2.4	>2.4	>2.4	>2.4
	1568	<0.3	1.05	1.50	2.40	2.40	2.40	2.40	2.25	>2.4	>2.4	>2.4	>2.4
	1569	<0.3	1.50	1.80	2.10	2.40	2.40	2.40	2.40	>2.4	>2.4	>2.4	>2.4
	1570	<0.3	0.90	1.95	2.25	2.40	2.40	2.40	2.40	>2.4	>2.4	>2.4	>2.4
1/3 Dose (0.66 ml)	1571	<0.3	0.90	1.65	2.10	2.10	2.40	2.40	2.40	>2.4	>2.4	>2.4	>2.4
	1572	<0.3	1.05	1.65	2.25	2.10	2.40	2.25	2.25	>2.4	>2.4	>2.4	>2.4
	1573	<0.3	0.90	1.80	2.40	2.40	2.40	2.40	2.40	>2.4	>2.4	>2.4	>2.4
	1574	<0.3	0.30	0.90	1.50	1.80	2.40	1.95	1.95	>2.4	>2.4	>2.4	>2.4
	**1575**	<0.3	0.60	2.10	2.10	2.10	2.10	1.80	2.40	>2.4	>2.4	>2.4	>2.4
1/9 Dose (0.22ml)	**1576**	<0.3	0.75	1.20	1.65	1.65	1.65	1.50	1.80	>2.4	>2.4	>2.4	>2.4
	1577	<0.3	1.05	1.50	1.65	1.50	2.25	1.65	1.95	>2.4	>2.4	>2.4	>2.4
	1578	<0.3	0.45	0.75	1.20	1.80	1.95	1.80	1.80	>2.4	>2.4	>2.4	>2.4
	**1579**	<0.3	0.30	<0.3	1.05	1.50	1.50	1.20	1.80	>2.4	>2.4	>2.4	>2.4
	**1580**	<0.3	0.30	0.75	1.20	1.35	1.35	1.05	1.65	>2.4	>2.4	>2.4	>2.4
Unvaccinated Controls	**1581**			<0.3	<0.3	0.30	1.05	<0.3	0.90	1.65	2.10	2.10	
	**1582**			<0.3	0.30	0.30	0.75	<0.3	0.90	1.65	1.80	1.95	2.10
	**1583**			<0.3	<0.3	0.30	1.35	<0.3	1.05	1.35	1.95	2.10	2.25

Supplementary Table 5b. VNT titres against A/IRN/22/2015 in vaccinated and unvaccinated cattle in the heterologous potency test vaccines (animal numbers in bold indicate cattle with foot lesions).

**Groups**	**Animal**	**Days post challenge**											
		**-21**	**-14**	**-7**	**0**	**1**	**2**	**3**	**4**	**5**	6	7	8
Full Dose (2ml)	1566	<0.3	0.60	1.05	1.20	1.05	1.05	1.50	1.35	1.95	2.10	>2.4	>2.4
	1567	<0.3	0.45	1.35	1.50	1.05	1.35	1.80	1.95	>2.4	>2.4	>2.4	>2.4
	1568	<0.3	0.90	0.75	1.20	1.20	1.20	1.35	1.35	>2.4	>2.4	>2.4	>2.4
	1569	<0.3	1.65	1.80	1.65	1.65	1.65	1.65	1.80	2.25	2.25	>2.4	>2.4
	1570	<0.3	0.30	1.20	1.35	1.35	1.65	2.10	1.95	>2.4	>2.4	>2.4	>2.4
1/3 Dose (0.66 ml)	1571	<0.3	0.30	0.90	1.35	1.20	1.35	1.50	1.50	2.25	>2.4	>2.4	>2.4
	1572	<0.3	1.20	0.90	1.05	1.05	1.20	1.20	1.80	>2.4	>2.4	>2.4	>2.4
	1573	<0.3	0.45	1.80	1.65	1.50	1.95	1.65	2.25	>2.4	>2.4	>2.4	>2.4
	1574	<0.3	<0.3	0.45	0.75	0.75	0.90	0.90	1.65	2.25	>2.4	>2.4	>2.4
	**1575**	<0.3	<0.3	0.60	0.60	0.75	1.05	0.75	1.50	>2.4	>2.4	>2.4	>2.4
1/9 Dose (0.22ml)	**1576**	<0.3	0.30	1.05	0.90	0.60	0.90	0.75	0.90	1.50	>2.4	>2.4	>2.4
	1577	<0.3	0.60	1.05	1.20	1.05	1.05	1.35	1.35	1.50	>2.4	>2.4	>2.4
	1578	<0.3	<0.3	<0.3	0.75	1.05	1.20	1.20	1.05	1.80	2.25	>2.4	>2.4
	**1579**	<0.3	<0.3	<0.3	0.30	0.90	1.35	0.75	1.35	1.95	2.25	>2.4	>2.4
	**1580**	<0.3	0.30	0.45	0.30	0.45	0.90	0.30	0.90	1.80	>2.4	>2.4	>2.4
Unvaccinated Controls	**1581**				<0.3	0.30	1.05	0.30	0.75	1.50	2.25	>2.4	>2.4
	**1582**				<0.3	0.30	0.90	0.30	1.35	1.80	2.25	>2.4	>2.4
	**1583**				<0.3	0.30	1.05	0.45	1.35	1.80	>2.4	>2.4	>2.4

Supplementary Table 6. Results of virus isolation and titration (VI; $\log_{10}$ PFU/ml) and viral RNA detected by real-time PCR (PCR; Cp values) from serum of vaccinated and unvaccinated cattle in the heterologous potency test vaccines (animal numbers in bold indicate cattle with foot lesions).

Group	Animal		0 DPC	1 DPC	2 DPC	3 DPC	4 DPC	5 DPC	6 DPC	7 DPC	8 DPC
Full dose (2 ml)	1566	PCR	-	-	-	-	32.37	-	-	-	-
		VI	-	-	-	-	-	-	-	-	-
	1567	PCR	-	-	-	-	32.24	-	-	-	-
		VI	-	-	-	-	-	-	-	-	-
	1568	PCR	-	-	-	-	-	-	-	-	-
		VI	-	-	-	-	-	-	-	-	-
	1569	PCR	-	-	-	31.92	-	-	-	-	-
		VI	-	-	-	-	-	-	-	-	-
	1570	PCR	-	-	-	32.02	32.45	-	-	-	-
		VI	-	-	-	-	-	-	-	-	-
1/3 dose (0.66 ml)	1571	PCR	-	-	30.29	-	-	-	-	-	-
		VI	-	-	-	-	-	-	-	-	-
	1572	PCR	-	-	29.87	32.03	-	-	-	-	-
		VI	-	-	-	-	-	-	-	-	-
	1573	PCR	-	-	-	-	-	-	-	-	-
		VI	-	-	-	-	-	-	-	-	-
	1574	PCR	-	-	-	32.09	-	-	-	-	-
		VI	-	-	-	-	-	-	-	-	-
	**1575**	PCR	-	-	-	30.83	-	-	-	-	-
		VI	-	-	-	-	-	-	-	-	-
1/9 dose (0.22 ml)	**1576**	PCR	-	31.56	29.53	28.68	31.73	-	-	-	-
		VI	-	-	-	-	-	-	-	-	-
	1577	PCR	-	-	-	32.55	31.94	-	-	-	-
		VI	-	-	-	-	-	-	-	-	-
	1578	PCR	-	-	-	-	-	-	-	-	-
		VI	-	-	-	-	-	-	-	-	-
	**1579**	PCR	-	28.66	30.81	29.81	29.72	-	-	-	-
		VI	-	-	-	-	-	-	-	-	-
	**1580**	PCR	-	30.81	30.40	26.93	30.96	-	-	-	-
		VI	-	-	-	-	-	-	-	-	-
Unvaccinated Controls	**1581**	PCR	-	17.1	14.79	14.56	25.5	-	-	-	†
		VI	-	>10.00	>10.00	>10.00	-	-	-	-	
	**1582**	PCR	-	19.68	17.70	19.47	29.00	31.94	-	-	-
		VI	-	>10.00	>10.00	>10.00	-	-	-	-	-
	**1583**	PCR	-	17.86	19.46	18.80	29.18	-	-	-	-
		VI	-	>10.00	>10.00	>10.00	-	-	-	-	-

Supplementary Table 7. Results of virus isolation and titration (VI; $\log_{10}$ PFU/ml) and viral RNA detected by real-time PCR (PCR; Cp values) from nose swabs of vaccinated and unvaccinated cattle in the heterologous potency test vaccines (animal numbers in bold indicate cattle with foot lesions).

Group	Animal		0 DPC	1 DPC	2 DPC	3 DPC	4 DPC	5 DPC	6 DPC	7 DPC	8 DPC
Full dose (2 ml)	1566	PCR	-	9.45	12.75	14.53	15.68	19.22	21.76	31.10	27.85
		VI	-	7.68	6.80	6.00	4.70	3.13	3.24	-	-
	1567	PCR	-	11.60	13.64	21.81	20.45	23.24	25.50	32.32	-
		VI	-	7.44	5.83	2.40	3.35	3.00	0.40	0.40	-
	1568	PCR	-	14.75	14.85	20.42	20.99	31.74	32.09	32.32	-
		VI	-	5.24	5.24	3.10	3.13	-	-	-	-
	1569	PCR	-	8.13	16.76	17.59	16.90	23.59	27.09	30.00	31.43
		VI	-	8.28	4.01	4.96	3.74	3.13	1.74	-	-
	1570	PCR	-	8.01	17.04	16.89	19.80	22.64	28.20	32.60	-
		VI	-	7.04	3.94	6.64	7.57	2.51	-	-	-
1/3 dose (0.66 ml)	1571	PCR	-	6.00	13.46	18.44	11.74	22.53	21.21	25.88	31.25
		VI	-	8.00	5.30	3.11	7.39	-	1.54	-	-
	1572	PCR	-	8.01	16.93	14.06	19.95	23.39	24.52	29.70	31.23
		VI	-	7.93	3.92	5.09	4.24	2.17	-	-	-
	1573	PCR	-	30.00	16.60	17.32	20.74	29.25	32.47	-	-
		VI	-	1.10	5.00	3.26	2.70	0.40	-	-	-
	1574	PCR	-	7.56	13.90	18.06	19.87	23.83	31.73	-	-
		VI	-	7.08	4.83	3.68	4.13	2.40	-	-	-
	**1575**	PCR	-	8.86	9.58	9.56	21.80	22.84	26.47	30.45	31.56
		VI	-	7.07	6.35	7.07	5.18	2.10	0.88	0.40	-
1/9 dose (0.22 ml)	**1576**	PCR	-	18.54	9.92	17.48	17.55	25.16	31.07	32.71	33.84
		VI	-	4.05	6.16	5.10	5.95	3.10	0.70	-	-
	1577	PCR	-	7.47	10.27	17.98	15.08	20.07	21.11	24.70	28.91
		VI	-	7.51	6.89	5.10	6.10	2.70	2.70	1.00	-
	1578	PCR	-	11.76	13.67	19.57	20.75	21.79	25.00	21.24	32.55
		VI	-	6.38	5.31	3.11	2.88	3.00	-	-	-
	**1579**	PCR	-	9.94	11.56	18.72	19.73	25.70	31.75	32.55	34.12
		VI	-	6.95	6.04	3.44	3.26	1.86	-	-	-
	**1580**	PCR	-	6.93	16.96	19.23	18.91	26.16	30.87	32.26	33.78
		VI	-	7.44	3.89	3.48	3.92	2.02	0.70	-	-
Unvaccinated Controls	**1581**	PCR	-	9.56	12.85	16.56	15.21	19.99	24.55	29.68	†
		VI	-	6.72	4.32	4.94	5.24	3.98	2.55	1.63	
	**1582**	PCR	-	8.09	13.87	17.64	19.94	22.62	25.84	28.75	27.24
		VI	-	7.13	3.89	3.77	3.76	3.00	1.54	-	-
	**1583**	PCR	-	20.81	14.25	17.72	18.03	23.80	29.50	27.02	27.71
		VI	-	3.68	4.88	3.88	3.90	2.15	0.70	0.40	-

Supplementary Table 8. Results of virus isolation and titration (VI; log_{10} PFU/ml) and viral RNA detected by real-time PCR (PCR; Cp values) from mouth swabs of vaccinated and unvaccinated cattle in the heterologous potency test vaccines (animal numbers in bold indicate cattle with foot lesions).

Group	Animal		0 DPC	1 DPC	2 DPC	3 DPC	4 DPC	5 DPC	6 DPC	7 DPC	8 DPC
Full dose (2 ml)	1566	PCR	-	25.61	30.58	30.83	29.90	30.46	31.89	-	-
		VI	-	2.69	1.51	1.81	0.88	0.40	-	-	-
	1567	PCR	-	31.18	29.65	30.59	31.72	32.02	32.39	-	33.76
		VI	-	0.40	1.10	0.40	-	-	-	-	-
	1568	PCR	-	29.32	29.91	30.46	29.65	29.81	31.70	-	-
		VI	-	1.00	1.86	-	-	0.88	-	-	-
	1569	PCR	-	-	-	31.39	31.74	32.08	-	-	-
		VI	-	-	-	-	0.70	-	-	-	-
	1570	PCR	-	29.00	-	31.17	32.07	31.80	-	-	-
		VI	-	0.88	-	-	-	-	-	-	-
1/3 dose (0.66 ml)	1571	PCR	-	30.93	31.21	31.23	30.52	31.63	32.61	32.45	-
		VI	-	0.88	-	-	-	-	-	0.40	-
	1572	PCR	-	30.94	31.38	24.87	29.22	30.60	31.74	32.59	-
		VI	-	-	-	2.56	1.00	-	-	-	-
	1573	PCR	-	-	31.22	31.75	30.20	-	-	-	-
		VI	-	-	-	-	-	-	-	-	-
	1574	PCR	-	24.68	30.80	29.84	31.45	31.59	-	-	-
		VI	-	3.30	-	0.70	-	-	-	-	-
	**1575**	PCR	-	30.04	31.11	31.29	30.99	31.85	31.87	32.72	33.46
		VI	-	1.00	1.00	0.40	0.40	-	-	-	-
1/9 dose (0.22 ml)	**1576**	PCR	-	30.41	31.22	31.66	31.42	-	31.94	-	32.04
		VI	-	0.88	-	-	0.70	-	-	-	-
	1577	PCR	-	-	-	31.45	31.63	30.78	-	32.58	-
		VI	-	0.40	-	-	-	-	-	-	-
	1578	PCR	-	-	23.70	29.89	32.00	31.48	-	-	-
		VI	-	-	1.93	1.57	-	0.40	-	-	-
	**1579**	PCR	-	30.92	24.19	29.26	30.01	29.27	30.88	32.73	-
		VI	-	2.10	3.20	1.40	1.70	0.40	-	-	-
	**1580**	PCR	-	-	24.45	31.84	29.22	31.74	32.40	-	-
		VI	-	-	3.61	0.70	2.06	-	-	-	-
Unvaccinated Controls	**1581**	PCR	-	27.64	19.44	19.36	18.19	27.58	28.17	30.50	†
		VI	-	2.79	3.69	4.72	4.65	1.44	0.40	-	
	**1582**	PCR	-	31.06	21.55	23.46	21.63	26.62	25.99	29.58	29.04
		VI	-	1.35	3.49	3.02	3.91	2.10	1.00	-	-
	**1583**	PCR	-	29.72	26.13	26.44	21.20	28.00	31.84	-	-

	VI	-	1.44	2.80	3.06	3.72	1.65	-	-	-

Supplementary Table 9a. Percentage inhibition in the Priocheck NS ELISA in the full dose protection test. Positive values above 50% inhibition are indicated in yellow vaccines (animal numbers in bold indicate cattle with clear or doubtful foot lesions).

Groups	Animal	0 DPC	1 DPC	2 DPC	3 DPC	4 DPC	5 DPC	6 DPC	7 DPC	8 DPC
Vaccine A_{22}/IRQ/64	1227	16	28	27	31	31	39	65	75	83
	**1228**	37	38	39	35	37	40	60	71	82
	**1229**	30	32	33	32	38	40	65	87	90
	**1230**	35	34	34	37	37	27	47	76	91
	**1231**	28	32	28	33	22	26	62	86	91
	1232	30	32	31	21	29	33	61	84	86
	**1233**	32	37	15	34	35	39	57	81	90
Vaccine A/MAY/97	**1234**	24	12	20	19	22	28	44	60	68
	1235	14	21	20	20	24	28	46	60	65
	1236	26	31	27	28	30	32	45	70	84
	**1237**	12	16	13	16	20	19	41	81	91
	1238	27	25	28	28	26	18	32	45	55
	1239	36	31	28	32	24	45	84	93	93
	1240	19	16	16	6	18	35	63	74	78
Unvaccinated Controls	**1241**	-2	3	-6	7	16	26	42	64	75
	**1242**	22	8	19	26	25	29	37	68	89
	**1243**	18	26	23	27	28	40	60	87	93

Supplementary Table 9b. Percentage inhibition in the Priocheck NS ELISA in the heterologous protection test vaccines (animal numbers in bold indicate cattle with foot lesions).

Groups	Animal	0 DPC	1 DPC	2 DPC	3 DPC	4 DPC	5 DPC	6 DPC	7 DPC	8 DPC
Full Dose (2ml)	1566	21	19	16	19	24	30	79	92	93
	1567	32	28	27	28	29	32	59	78	85
	1568	15	17	14	15	18	13	32	55	70
	1569	37	40	42	40	41	35	44	64	80
	1570	41	40	41	42	43	61	81	90	93
1/3 Dose (0.66 ml)	1571	27	27	25	32	28	32	59	76	80
	1572	52	41	43	41	36	38	73	91	78
	1573	11	6	9	11	11	15	50	74	77
	1574	34	33	28	32	26	30	41	54	83
	**1575**	27	23	20	24	26	30	71	87	92
1/9 Dose (0.22ml)	**1576**	30	27	24	26	27	36	59	86	92
	1577	24	23	21	27	26	32	55	85	90
	1578	35	40	39	43	43	47	52	70	87
	**1579**	44	46	43	49	42	52	67	84	91
	**1580**	23	21	19	29	29	39	54	73	84
Unvaccinated Controls	**1581**	24	24	19	20	22	31	51	77	NA
	**1582**	31	26	27	34	32	41	63	84	90
	**1583**	29	23	20	28	22	34	44	77	89
